# Notice to Foreign Medical Journals

**Published:** 1858-09

**Authors:** 


NOTICE TO FOREIGN MEDICAL JOURNALS.—The *Dublin Medical Press*, *Gazette Médicale*, the July number of the *Glasgow Medical Journal*, the *Medical Times and Gazette* for July 3d, the second number of the *Journal de la Physiologie de l'Homme èt des Animaux*, and Beale's *Archives of Medicine* have not reached us.

